# Monitoring Fetal Heart Rate during Labor: A Comparison of Three Methods

**DOI:** 10.1155/2017/8529816

**Published:** 2017-03-14

**Authors:** Tammy Y. Euliano, Shalom Darmanjian, Minh Tam Nguyen, John D. Busowski, Neil Euliano, Anthony R. Gregg

**Affiliations:** ^1^Department of Anesthesiology, University of Florida College of Medicine, Gainesville, FL, USA; ^2^Department of Obstetrics and Gynecology, University of Florida College of Medicine, Gainesville, FL, USA; ^3^OBMedical, Jonesville, FL, USA; ^4^Winnie Palmer Hospital for Women & Babies, Orlando, FL, USA

## Abstract

The purpose of the study was to compare the accuracy of a noninvasive fetal heart rate monitor with that of ultrasound, using a fetal scalp electrode as the gold standard, in laboring women of varying body habitus, throughout labor and delivery. Laboring women requiring fetal scalp electrode were monitored simultaneously with the investigational device (noninvasive fetal ECG), ultrasound, and fetal scalp electrode. An algorithm extracted the fetal heart rate from the noninvasive fetal ECG signal. Each noninvasive device recording was compared with fetal scalp electrode with regard to reliability by positive percent agreement and accuracy by root mean squared error. Seventy-one women were included in this analysis. Positive percent agreement was 83.4 ± 15.4% for noninvasive fetal ECG and 62.4 ± 26.7% for ultrasound. The root mean squared error compared with fetal scalp electrode-derived fetal heart rate was 4.8 ± 2.0 bpm for noninvasive fetal ECG and 14.3 ± 8.2 bpm for ultrasound. The superiority of noninvasive fetal ECG was maintained for stages 1 and 2 of labor and increases in body mass index. Compared with fetal scalp electrode-derived fetal heart rate, noninvasive fetal ECG is more accurate and reliable than ultrasound for intrapartum monitoring for stages 1 and 2 of labor and is less affected by increasing maternal body mass index. This confirms the results of other workers in this field.

## 1. Introduction

Fetal well-being is assessed through electronic monitoring of uterine activity and fetal heart rate (FHR) patterns. These signals can be obtained noninvasively, traditionally through tocodynamometry and Doppler ultrasound (US), or invasively with an intrauterine pressure catheter (IUPC) and fetal scalp electrode (FSE). The latter methods require ruptured membranes and entail some small risk of infection [1, 2] and bleeding [3] but generally suffer less signal loss and provide additional information: the IUPC provides quantitative intrauterine pressure, and the fetal ECG can be obtained from the FSE.

International Federation of Gynecology and Obstetrics recommendations for FHR monitoring [4] include “that the baseline and the variability may be clearly read off at least 80% of the time.” That target is often not reached with US [5, 6], particularly in the obese parturient [7].

An alternative noninvasive method entails detecting both uterine activity [electrohysterogram (EHG)] and FHR [noninvasive fetal ECG] via electrodes located on the maternal abdomen. This technology is less dependent on proximity of the sensor to the target and therefore functions regardless of the patient's body habitus.

We previously demonstrated the superiority of EHG over tocodynamometry for uterine activity monitoring, using IUPC as the gold standard [8]. For the current study, we used a similar methodology to compare the abdominal fetal ECG (afECG) with US, using FSE as the gold standard.

## 2. Materials and Methods

This study is an analysis of the FHR data from an unpublished larger data collection, using only those patients monitored simultaneously with all three FHR devices. It was conducted at two Florida hospitals: UF Health, the University of Florida's teaching hospital (Gainesville, FL), and Winnie Palmer Hospital for Women & Babies (Orlando, FL). The protocol was approved by the Institutional Review Boards at both institutions (UF# 346-2010, WP# 13.153.09) and each subject provided written, informed consent. Adult women admitted to the labor and delivery suites at term (≥37 weeks' gestation), in active labor with a singleton fetus in cephalic presentation, without bleeding, uterine scar, or evidence of chorioamnionitis, and with FSE in place for obstetric indication (as determined by the attending obstetrician) were eligible for inclusion.

**Figure 1:**
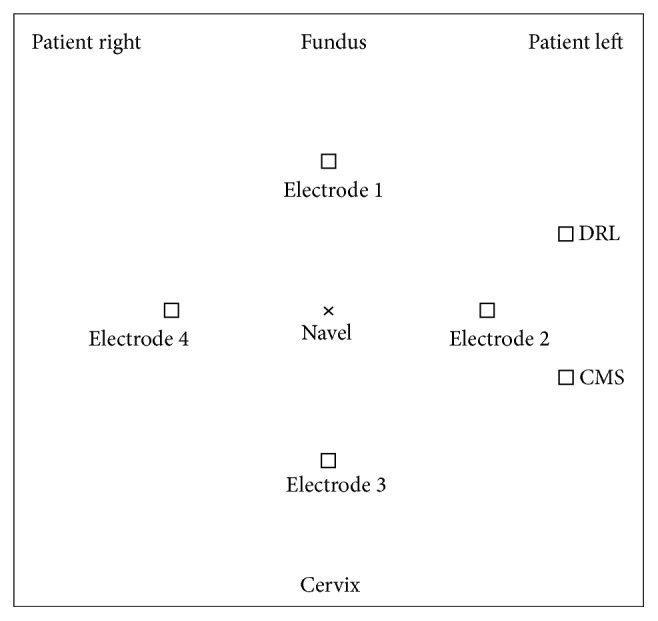
Location of electrodes.

Following skin preparation by gentle rubbing with abrasive gel (OneStep AbrasivPlus, Liquimedics Pty Ltd., Germany), six 3-cm^2^ Ag/AgCl_2_ electrodes (T-00-S; Ambu; Glen Burnie, MD) were attached to the maternal abdomen (Figure 1). The electrodes were connected to the amplifier in a monopolar fashion with common reference and common mode rejection leads on the left side of the patient's abdomen to reduce 60 Hz environmental noise. Electrode positions were modified slightly for each patient, as required by the location of the tocodynamometer and US FHR monitor. Impedance of each electrode was measured (as compared with the reference) (General Devices 10 Hz EIM-105 Prep-Check; Ridgefield, NJ). Skin preparation was repeated as needed at each site until the measured impedance was below 10 kΩ where possible.

In addition to the experimental system, data from each patient included FHR from the maternal-fetal monitor: US (Corometrics 250 series, GE Medical Systems, Waukesha, WI) and FSE (Corometrics at UF Health, and Avalon FM50, Philips Healthcare; Andover, MA at Winnie Palmer) sampled at 4 Hz with 8-bit resolution, upsampled to 8 Hz to match other signals from the monitors. These cardiotocographs reported the US- and FSE-derived FHR.

The signals recorded from the electrodes were fed to the custom built, four-channel high-resolution, low-noise unipolar amplifier based on the TI ADS family of ECG/EEG amplifiers. All four signals were measured with respect to the reference electrode. The amplifier design employed driven right-leg circuitry (derived from a combination of the four channels) to reduce common mode noise. The amplifier 3 dB bandwidth was 0.05 to 250 Hz. Data from four abdominal channels were sampled at 500 Hz with 24-bit resolution.

Because the muscle activity of the uterus (EHG) differs in frequency from the maternal and fetal heart rates, a simple frequency-selective filtering technique can separate the signals, allowing for the contraction-monitoring algorithm and the FHR algorithm to be largely independent. The maternal and fetal heart rates, however, overlap in frequency. The Mermaid algorithm is in a class called blind source separation or independent component analysis. These algorithms allow for the separation of overlapping signals as long as they are created by independent sources. The algorithm employed requires at least one electrode for each independent source and uses small differences in each electrode and correlation between the channels and sources, to separate the mixed signals. This occurs in real time [9].

Four abdominal electrode signals are input to the system from the hardware described above. These signals are first preprocessed and filtered with a bandpass filter between 1 and 30 Hz to remove noise and the EHG signal. Next, the Mermaid algorithm finds the four largest independent signal sources in the mixed signals (e.g., maternal ECG, fetal ECG, breathing, muscle noise, and other noises). A second algorithm then selects the channel with properties expected in the fetal ECG. A trust factor reports how well the system was able to extract the desired signals.

Data from the FSE, US (using a second electronic fetal monitoring unit), and afECG were collected simultaneously via a laptop computer. The data collector was instructed to attempt to reposition the US to obtain a reliable signal in all subjects. Clinicians were blinded to all but the FSE output for FHR monitoring and intervention.

Reliability was assessed by the positive percent agreement (PPA), the percentage of time the noninvasive device (US or afECG) generated FHR within 10% of the FHR from the FSE. FHR estimation of afECG utilized a 10-beat average to approximate the averaging from the other devices. This was calculated for each subject and averaged. Accuracy of each FHR output was estimated using the root mean squared error (RMSE), the instantaneous FHR differences between comparator and FSE. Subject populations at each study site were compared using a paired *t*-test with a significance level of 0.025.

## 3. Results

Patient characteristics did not differ between sites (Table 1).

The average PPA for afECG, 83.4%, exceeded that for US (62.4%, *p* < 0.0001, Table 2). That superiority persisted in both first (*p* < 0.0001) and second (*p* < 0.003) stages of labor for all subjects. Furthermore, afECG was more accurate, with a mean RMSE of 4.8 bpm compared to 14.3 for US (*p* < 0.0001). In obese parturients (BMI > 30 kg/m^2^), the afECG again outperformed US in both PPA (84.4% versus 58.1%, *p* < 0.0001) and accuracy (4.8 versus 15.6 bpm, *p* < 0.0001). Furthermore, afECG showed no drop-off in performance between normal weight (PPA 81.0%  ± 17.2%; RMSE 4.9 ± 1.9 bpm) and obese subjects (PPA 84.4 ± 14.6%; RMSE 4.8 ± 2.1 bpm), while US performance was adversely affected by obesity: normal weight PPA 73.2%  ± 25.2%; RMSE 11.0 ± 7.2 bpm; obese PPA 58.1%  ± 25.8%; RMSE 15.6 ± 8.1 bpm.

## 4. Discussion

Doppler US is the most common method for continuous FHR monitoring and functions adequately in most situations. Its frequent regions of dropout and occasional confusion with maternal heart rate [10] complicate interpretation in the setting of a nonreassuring tracing. When external monitoring is unreliable, clinicians may artificially rupture membranes to place more dependable internal monitors. This increases the duration of ruptured membranes and risk of infection.

Failure of external US monitoring is more common in the obese population [7], comprising nearly one-third of women of child-bearing age [11]. These patients are more likely to experience complications [12–14], require internal monitoring [7], have prolonged first-stage labor [15], and undergo cesarean delivery [14, 16, 17]. The reason for the increased cesarean rate is likely multifactorial, including the slow pace of cervical dilation and the concomitant increased number of cervical examinations and need for internal monitoring, resulting in a higher rate of chorioamnionitis, which itself increases the cesarean delivery rate [18].

An alternative external monitoring system that provides reliable FHR and uterine activity regardless of maternal size may improve outcomes. In addition, the continuous display of maternal heart rate will reduce maternal-fetal heart rate confusion incidents.

Cohen et al. [19] compared US, FSE, and fECG from the maternal abdomen (afECG) via the AN24 system (Monica Healthcare Ltd., Nottingham, UK) in 75 laboring women with a protocol similar to that reported here. PPA was reported as the percentage of time the external monitor reported FHR within 10% of that derived from the FSE and was superior for the afECG device (81.7% versus 73% for US). This superiority persisted in analysis of both first- and second-stage labor (PPA afECG 84.9% and 71.9% versus US 74.7% and 61.7%, for first and second stage, resp.). The afECG also demonstrated improved accuracy with RMSE of 5.3 ± 2.4 bpm versus 10.9 ± 5.8 bpm for US.

The success of their afECG compares favorably with our results, though US underperformed in our study. In both studies, FSE was placed for obstetric indication. Cohen states, “in all cases [FSE was placed] because the external tracing was abnormal.” Whether abnormal in this case includes high dropout is unclear. Although we did not record the indication in our study, subjects were more likely to have an unacceptable rate of dropout with US, thus biasing our results against that device. Furthermore, in both studies, subjects were monitored clinically by FSE. Although both protocols specified adjustments to US when it failed, it is possible that US was not optimally used during the study. Regardless, the PPA comparison of afECG with FSE is valid and proves the utility of afECG in the population where US fails.

Graatsma et al. [20] found no impact of increasing body mass index (BMI) on afECG signals with the AN24 system. Their cohort was 20- to 42-week, nonlaboring pregnant women monitored during sleep and therefore differs substantially from the active labor environment of this report.

Cohen and Hayes-Gill [21] looked specifically at the impact of BMI on the accuracy and reliability of external monitoring. In a secondary analysis of 74 parturients monitored simultaneously by all three methods (US, FSE, and AN24), they found no effect of maternal obesity on the performance of their system, while US performance “degraded directly with maternal size.” In their study, nine subjects with BMI > 40 had PPA of 81.4 ± 23.8% for afECG. This compares to our results of 86.1 ± 15% on 19 such patients.

In summary, we found that afECG is superior to US in both reliability and accuracy when compared to FSE in all subjects and in the obese subset. This may have clinical implications for the assessment of fetal well-being, particularly in the obese subjects, where US frequently fails to provide an adequate FHR trace.

## Figures and Tables

**Table 1:** Demographics comparison between sites.

Demographic variables	Total (*n* = 71)	UF Health (*n* = 10)	Winnie Palmer (*n* = 61)	*p*
Age (years)	27.8 ± 6.2	25.2 ± 5.6	28.3 ± 6.2	0.15
Gestational age (weeks)	39.1 ± 1.3	39.5 ± 2.0	39.0 ± 1.1	0.23
Body mass index	35.1 ± 8.3	38.2 ± 8.7	34.6 ± 8.2	0.22

*Monitoring uterine activity during labor*.

**Table 2:** Performance of abdominal fetal ECG and ultrasound compared to fetal scalp electrode.

All stages	All subjects			Obese subjects: BMI ≥ 30		
	Ultrasound	afECG	*p*	Ultrasound	afECG	*p*
	(*n* = 71)			(*n* = 51)		
PPA (%)	62.4 ± 26.5	83.4 ± 15.4	<0.0001	58.1 ± 25.8	84.4 ± 14.6	<0.0001
RMSE (bpm)	14.3 ± 8.2	4.8 ± 2.0	<0.0001	15.6 ± 8.1	4.8 ± 2.1	<0.0001

Stage 1	(*n* = 48)			(*n* = 36)		
PPA (%)	61.3 ± 29.6	86.3 ± 14.7	<0.0001	55.6 ± 28.8	79.1 ± 13.3	<0.0001
RMSE (bpm)	13.6 ± 7.9	5.0 ± 2.0	<0.0001	15.5 ± 8.1	4.9 ± 2.0	<0.0001

Stage 2	(*n* = 23)			(*n* = 15)		
PPA (%)	64.5 ± 18.5	77.5 ± 15.1	<0.003	64.0 ± 15.3	79.1 ± 13.3	<0.007
RMSE(bpm)	15.8 ± 8.4	5.0 ± 2.0	<0.0001	15.7 ± 7.9	4.9 ± 2.0	<0.0002

*Monitoring uterine activity during labor*.
